# Adipokines and Insulin Resistance in Young Adult Survivors of Childhood Cancer

**DOI:** 10.1155/2016/6349134

**Published:** 2016-04-24

**Authors:** Eryk Latoch, Katarzyna Muszynska-Roslan, Agata Panas, Anna Panasiuk, Malgorzata Sawicka-Zukowska, Beata Zelazowska-Rutkowska, Ewa Zabrocka, Maryna Krawczuk-Rybak

**Affiliations:** ^1^Department of Pediatric Oncology and Hematology, Medical University of Bialystok, 15-274 Bialystok, Poland; ^2^Department of Pediatric Laboratory Diagnostics, Medical University of Bialystok, 15-274 Bialystok, Poland; ^3^Student's Scientific Society by the Department of Pediatric Oncology and Hematology, Medical University of Bialystok, 15-274 Bialystok, Poland

## Abstract

We examined the association between adipokines (leptin, adiponectin, and resistin), radiotherapy, measurement of body fat, and insulin resistance among young adult survivors of childhood cancer (CCS).* Materials and Methods.* Seventy-six survivors were included (mean age 24.1 ± 3.5 years). Insulin resistance (IR) was calculated using the homeostasis model assessment (HOMA-IR). The serum levels of adipokines were assayed by immunoassays. Fat mass was evaluated by DXA.* Results.* Mean adiponectin level and mean body FAT were higher in the examined females than in males (10009 ± 6367 ng/mL versus 6433 ± 4136 ng/mL, *p* < 0.01; 35.98 ± 9.61% versus 22.7 ± 7.46%, *p* < 0.001). Among CCS, one of 75 patients met the criteria of insulin resistance, and in 14 patients there was impaired fasting glucose. The multiple regression model for females showed that leptin/adiponectin ratio (LA ratio) significantly affected HOMA-IR (increase of 0.024 per each unit of LA ratio; *p* < 0.05). Radiotherapy had no effect on serum adipokines and IR.* Conclusion.* The observed results support the hypothesis that adiponectin might be associated with insulin resistance and it can not be ruled out that changes in the mean level of adiponectin per FAT mass or leptin/adiponectin ratio may precede the occurrence of insulin resistance in the future.

## 1. Introduction

In recent years, advances in diagnosis and treatment have caused a significant increase of the number of young adults who experienced cancer in childhood [1, 2]. The latest available study conducted in US shows that more than 60% of survivors suffer from at least one chronic condition and nearly 30% of former patients developed severe or life-threating sequelae [3]. Recent reports emphasize that cancer survivors have an increased prevalence of overweight, metabolic syndrome and may be at risk of developing insulin resistance (IR) [4–7].

The underlying processes for IR are multifactorial and still not fully explained. It has been already noted that radiotherapy, especially cranial radiation, plays a significant role in the development of obesity and insulin resistance [8]. Other unfavorable factors include the use of glucocorticoids, decreased physical activity, and poor dietary habits.

Leptin, adiponectin, and resistin are peptide hormones, which might have a potential role in the mechanism leading to insulin resistance [9]. These adipokines are secreted by adipose tissue and regulate, among other things, glucose and lipid metabolism [10]. Hyperleptinemia and hypoadiponectinemia have been associated with increased risk of insulin resistance, overweight, and diabetes mellitus in adult patient [11]. Likewise, high level of resistin has been postulated to be involved in insulin dysfunction.

The aim of our study was to evaluate the relationship between plasma concentrations of adipokines, radiotherapy, measurement of body fat, and insulin resistance in young childhood cancer survivors (CCS).

## 2. Material and Methods

### 2.1. Study Population

The invitation for a follow-up visit had been sent to 150 former patients of the Department of Pediatric Oncology and Hematology of Medical University of Bialystok, of whom 75 responded positively (43 men and 32 women). Among those 29 (38%) were treated for leukemia, 27 (36%) for lymphoma, and 19 (26%) for solid tumors (except central nervous system tumors). The mean age at the time of study was 24.11 ± 3.5 years and the average time from the end of treatment was 11.81 ± 5.2 years (range 1–23.5). The characteristics of patients are shown in Table 1. All patients were treated according to international protocols approved by Polish Pediatric Leukemia/Lymphoma Group and Polish Pediatric Solid Tumor Group (acute lymphoblastic leukemia: BFM ALL 90 and 95, ALL IC 2002; lymphoma: BFM NHL 90, LMB, BFM NHL 04, MVPP/B-DOPA, EURONET; nephroblastoma: SIOP protocols; neuroblastoma: PACE, SIOP HRG). All of them were in complete continuous remission. Seventy-two percent of the participants were treated with radiation therapy (RT) (cranial CRT and/or local RT). Hematological stem cell transplantation procedure (BMT) was performed in 6 patients treated previously for leukemia (4) and lymphoma (2); all of them received total body irradiation (TBI). A written informed consent was obtained from all subjects and the Ethics Committee of the Medical University of Bialystok approved the study.

### 2.2. Anthropometric Measurements of Body Composition

An overview of all medical records was performed to obtain data concerning age, type of diagnosis, and treatment. All patients underwent a clinical examination and anthropometric measurements during follow-up visit (standard techniques were used). Weight was measured on a digital scale (Seca, Germany) and height was taken using a Martin anthropometer. In order to classify overweight and obesity we used Body Mass Index (BMI) International Classification by WHO. It was calculated as weight in kilograms divided by height in meters squared (kg/m^2^). Waist-hip ratio (WHR) was assessed as a marker for visceral fat (waist circumference divided by hip circumference). Fat body mass (FAT) (g), FAT percentage (%), and lean body mass (lean) were evaluated by dual-energy X-ray absorptiometry (DXA) (DPX-L, GE-Healthcare Lunar, Madison, WI). The lean/FAT ratio (L/F) was used to determine differences related to the percentage of the individual components of body weight.

### 2.3. Biochemical Analysis

All laboratory tests were performed following an eight-hour overnight fast. Blood samples were stored frozen at −80°C. Commercial immunoassays were used to measure the serum insulin, leptin, adiponectin, and resistin levels (R&D System, Inc., Quantikine®). Insulin resistance was evaluated using the homeostasis model assessment (HOMA-IR) according to the following formula: serum insulin (*μ*IU/mL) × plasma glucose (mmol/L)/22.5 [12]. HOMA-IR above 2.86 was considered abnormal as previously described [13, 14]. Leptin/adiponectin ratio (LA) was calculated. Due to the fact that concentration of adipokines depends to a large extent on the body fat mass, we calculated both leptin and adiponectin per kilogram fat mass. At the time of study patients were not taking drugs that might affect the level of insulin. The whole study group was divided into two subgroups: obese/overweight and nonobese/nonoverweight patients. All laboratory results were compared with the control group that consisted of 49 healthy counterparts.

### 2.4. Analysis

Statistical analysis was performed with STATA 11.0 version (StatCorp, College Station, Texas, USA). Data were expressed as means ± standard deviation (SD) or median and quartiles when appropriate. In the univariate analysis Fisher exact test and *χ* ^2^ test were used, whereas continuous variables were compared with the Wilcoxon rank-sum test or Student's* t*-test. Multivariate regression models were used to examine the association between HOMA-IR and the independent variables, which potentially might influence insulin resistance. The number of variables was adapted to the size of the group. A *p* value < 0.05 was considered significant.

## 3. Results

### 3.1. Characteristics of Study Participants

Demographic characteristics of the participants are provided in Table 1. There were no significant differences in the age at study and time after the end of treatment of the patients according to neither sex nor group of diagnosis. Among the whole study group, 50 patients received radiotherapy.

### 3.2. Insulin Resistance

Characteristics for men and women are presented in Table 2. HOMA index revealed that in 1 out of 75 patients insulin resistance was present and in fourteen (19%) patients there was impaired fasting glucose (≥5.6 mmol/L). Six of these (46%) patients had BMI above 25. The level of fasting glucose was statistically significantly higher in men in comparison with women (mean, 5.2 versus 4.7 mmol/L; *p* < 0.01). However, fasting insulin levels and HOMA-IR did not differ by gender. When the whole group was split depending on the BMI, a mean HOMA-IR and insulin level were found higher among the subjects with abnormal Body Mass Index in comparison with those with BMI within the normal range. In contrast, while compared to adiponectin per gram fat mass, an inverse link was noticed (Table 3).

### 3.3. Adipokines and Anthropometric Features

All gender differences in analyzed parameters are shown in Table 2. Adiponectin blood level and body FAT (total and percent) were statistically significantly higher in the examined females than in males (10009 ± 6367 versus 6433 ± 4136; *p* < 0.01), though adiponectin per body FAT in gram did not reveal any differences according to sex. Leptin, leptin per body FAT in gram, resistin, and LA ratio (leptin/adiponectin) did not differ between females and males. It was also found that lean mass was higher among men (*p* < 0.001). Due to the fact that women had greater body FAT and lower lean, the L/F ratio (lean/FAT) in men was nearly twice as high (*p* < 0.001). There were no differences between analyzed variables in the study group in comparison with the control group.

The body height, weight, and BMI were higher among male subjects (*p* < 0.05). In view of the fact that just one patient met the criteria of insulin resistance the whole group was divided according to the BMI in order to evaluate the parameters associated with lipid metabolism in obese and overweight patients with comparison with those with normal Body Mass Index.

Despite the fact that overweight patients presented statistically higher insulin levels and HOMA-IR we did not find the differences in adiponectin, leptin, and resistin levels between overweight and nonobese survivors. Descriptive data are shown in Table 3.

Analysis by gender (Table 2) showed that males had statistically significant higher BMI, WHR, lean, and lower FAT than in females; hence men had greater lean/FAT ratio. These groups did not differ from each other due to the concentration of adipocytokines.

### 3.4. Radiotherapy

We classified the patients into two groups regarding history of radiotherapy.  Table 4 presents a comparison of all analyzed parameters (directly or indirectly) related to the risk of insulin resistance in each group. This analysis did not show any differences between the subjects. Similarly, analysis of the same parameters between CRT and no CRT patients and CRT patients and those who received local radiotherapy did not reveal any significant differences (*p* < 0.05).

### 3.5. Correlates of Insulin Resistance

Univariate analysis demonstrated no correlation between HOMA-IR and the variables listed in the tables presented. Sex and radiotherapy were also taken into consideration. The multiple regression models were developed regarding each variable that might influence insulin resistance. Potential confounding variables included insulin, glucose, body FAT, lean, adiponectin, leptin, LA ratio, resistin, BMI, WHR, and lean/FAT ratio. The best models (with the highest coefficient of determination) of the functional relationship between HOMA-IR and independent variables are presented in Table 5. The model created for females showed that LA ratio significantly affected HOMA-IR (increase of 0.024 in HOMA-IR per each unit of LA ratio; *p* < 0.05). Interestingly in the same BMI model for males was found to have statistically significant influence on HOMA-IR (increase of 0.099 in HOMA-IR per each unit of BMI; *p* < 0.05).

## 4. Discussion

Most of the studies carried out on childhood cancer survivors demonstrate that this is a specific group of patients with increased susceptibility to the occurrence of a number of late sequelae. Endocrine disorders, including abnormal biological response of cells to normal insulin concentrations, are among the most common complications. Even though previous studies of CCS noticed a high prevalence of insulin resistant, we found only one patient with IR (1.3%) [9, 15]. One of the reasons explaining this discrepancy might be the fact that the study group consisted mostly of young adults whereas according to the available literature secondary IR increases with age and it is mainly influenced by lifestyle diseases [16]. Furthermore, the time elapsed since the end of treatment might have been too short to note the insulin metabolism impairment.

In recent years, there has been an increasing amount of literature focusing on insulin resistance and obesity in adults and children in general [17]. It has been reported that serum adiponectin level is negatively linked to the occurrence of metabolic syndrome and its concentration to the fat mass is inversely proportional. In the case of level of leptin relationship to body fat and the prevalence of metabolic syndrome it is quite the opposite [18–22]. Interestingly, Moschovi et al. reported that low plasma adiponectin and high leptin and resistin levels are present in acute lymphoblastic leukemia (ALL) at the time of diagnosis [23]. The authors emphasize the potential role of adipocytokines as noninvasive markers that may correlate with the stage of disease and response to treatment. Petridou et al. have showed that elevated serum adiponectin, but not leptin levels, might be independently associated with both childhood Hodgkin lymphoma and non-Hodgkin lymphoma incidence, as well as poor prognosis [24, 25]. Unfortunately, we could not make such an analysis due to the lack of data on the level of cytokines at diagnosis. Lower adiponectin level was found in males than in females but no differences were found in leptin and resistin levels.

For the first time the relationship between adipocytokines, insulin resistance, and measures of body fat mass in acute lymphoblastic leukemia survivors was described by Tonorezos et al. The author's attention was mainly focused on understanding how and when insulin resistance develops in long-term survivors of childhood ALL. They reported that women who were treated with cranial radiotherapy (CRT) had higher leptin levels, increased body fat, and lower adiponectin levels in comparison with males [9, 14, 15]. Similarly, our study has shown a significantly greater body FAT and FAT percentage in women than in men, though it was not associated with cranial radiotherapy. Contrary to our expectations, adiponectin blood level was found to be statistically higher in females than in males; however, after adjusting adiponectin level to body fatness, differences between genders were not observed. It seems that concentration of adiponectin remained proportional to body fat, despite the fact that fatness was higher in the female group. Likewise, we did not find leptin differences between groups. In this study, the resistin level was assessed as well, yet neither differences among groups nor any correlations between variables analyzed were found. The impact of radiation (especially CRT and high-dose radiotherapy) on weight gain in CCS was widely investigated [26, 27]. The authors hypothesized that RT might affect cellular metabolism and hence lead to abnormal cytokine production. However, our study did not confirm this relation. This could be due to the heterogeneity of the study group, use of various treatment protocols, and small sample size.

Moreover, we found statistically significant differences in adiponectin per gram FAT mass between overweight and obese subjects in comparison with nonoverweight and nonobese subjects. Lower mean adiponectin per gram FAT mass was seen in patients with BMI ≥ 25. When we divided patients by BMI, we also found that mean insulin level and mean HOMA-IR were significantly higher in subjects with BMI ≥ 25 in comparison with nonoverweight and nonobese subjects. Some previous researches documented that mean adiponectin per gram FAT mass was lower in the IR patients in comparison with the non-IR patients. We noticed a similar pattern when the patients were divided according to BMI rather than IR.

Some studies carried out in recent years also emphasize the potential role of gut hormones in the development of obesity (especially ghrelin and peptide YY). Ghrelin acts mainly via growth hormone receptors and stimulates food intake in humans. In contrast, PYY increases satiety and its effect is mediated by Y2 receptors which are also located in central nervous system [28, 29]. To our knowledge there are no studies evaluating the role of gut hormones in the pathogenesis of obesity and insulin resistance in childhood cancer survivors. Antineoplastic treatment (i.e., cranial or abdominal radiotherapy) may affect gut-brain axis function and visceral sensitivity by neurotransmitters and the other factors that regulate gastrointestinal absorption. In our opinion further research on gut hormones and their role in the development of obesity in CCS may allow for better understanding of the mechanism leading to insulin resistance.

The limitations of this study include a short observation period and a limited cohort size, which was on the grounds of a single center involved in this study. Due to the lack of data output time analysis was not feasible. Furthermore, the heterogeneity of the study group and the use of different treatment protocols did not allow the inference of causality.

The available data concerning insulin resistance in childhood cancer survivors are ambiguous. This study did not indicate that there is a high prevalence of IR among CCS. However, we found little evidence that this group of patients might present an increased risk of developing insulin disturbances that may result in the future in diabetes or metabolic syndrome. At present, there are still few studies in this field and it seems that further study is warranted.

The observed results support the hypothesis that adiponectin might be associated with insulin resistance and it cannot be ruled out that changes in the mean level of adiponectin per FAT mass or leptin/adiponectin ratio may precede the occurrence of insulin resistance in the future. However, it is still unclear and requires further studies.

## Figures and Tables

**Table 1:** Patient characteristics. Data are *n* (%) and mean ± SD. Four patients received both CRT and TBI.

	Total	Leukemia	Lymphoma	Solid tumor	*p*
Patients	75	29 (39)	27 (36)	19 (25)	0.33
Sex					
Male	43 (57)	11 (38)	14 (52)	8 (42)	0.44
Female	32 (43)	18 (62)	13 (48)	11 (58)	0.39
Age at study (years)	24.11 ± 3.5	24.19 ± 3.76	25.4 ± 4.11	22.87 ± 3.39	>0.05
BMI (kg/m^2^)	23.68 ± 4.01	23.77 ± 3.68	23.77 ± 3.68	22.77 ± 4.83	>0.05
Follow-up after treatment (years)	11.81 ± 5.2	12.11 ± 5.12	11.48 ± 4.07	11.86 ± 6.79	>0.05
Radiotherapy	50 (72)	19 (69)	20	11	0.23
Cranial (CRT)	19 (25)	19 (65)	—	—	—
Local	29 (39)	—	18 (67)	11 (58)	0.19
Total body Irradiation (TBI)	6 (8)	4 (14)	2 (7.4)	—	0.41

**Table 2:** Childhood cancer survivors' characteristics, by gender. Data are median and quartiles (1q; 3q) or mean ± SD.

	Total		Females		Males		*p* value
	*n* = 75		*n* = 32		*n* = 43		
	Mean ± SD	Median (q1; q3)	Mean ± SD	Median (q1; q3)	Mean ± SD	Median (q1; q3)	
Age (years)	24.11 ± 3.51	23.59 (21.46; 25.99)	23.88 ± 3.99	23.75 (20.60; 25.95)	24.29 ± 3.131	23.55 (22.05; 26.01)	0.476
Time from treatment completion (years)	11.81 ± 5.19	12.15 (7.35; 15.50)	11.26 ± 4.94	12.15 (6.62; 14.83)	1224 ± 5.40	12.00 (7.50; 16.50)	0.420
Weight (kg)	71.05 ± 14.99	70.00 (61.00; 80.40)	62.72 ± 12.40	61.05 (54.05; 69.70)	77.26 ± 13.78	77.40 (66.10; 83.70)	<0.001
Height (m)	1.73 ± 0.09	1.72 (1.67; 1.80)	1.66 ± 0.07	1.67 (1.63; 1.72)	1.77 ± 0.07	1.78 (1.72; 1.82)	<0.001
BMI (kg/m^2^)	23.68 ± 4.01	22.94 (20.96; 25.67)	22.59 ± 3.73	21.80 (19.54; 25.07)	24.49 ± 4.06	24.29 (21.74; 26.61)	0.025
WHR	0.86 ± 0.13	0.83 (0.79; 0.92)	0.83 ± 0.17	0.80 (0.71; 0.83)	0.88 ± 0.09	0.86 (0.83; 0.93)	<0.001
FAT (g)	21063 ± 11367	17361 (12779; 27228)	26605 ± 14112	23390 (17043; 36393)	18368 ± 8772	15753 (12380; 24093)	0.025
FAT (%)	27.04 ± 10.28	26.50 (19.80; 34.20)	35.98 ± 9.61	37.25 (29.48; 41.58)	22.70 ± 7.46	21.20 (16.75; 28.85)	<0.001
Lean (g)	50644 ± 10237	52163 (41666; 58885)	38921 ± 4133	39487 (36211; 41669)	56347 ± 6855	56244 (51934; 61920)	<0.001
Lean/FAT	3.14 ± 1.76	2.64 (1.83; 3.86)	1.90 ± 1.09	1.58 (1.16; 2.36)	3.75 ± 1.71	3.53 (2.33; 4.71)	<0.001
Insulin (*µ*IU/mL)	9.49 ± 15.09	6.39 (3.69; 10.70)	8.37 ± 5.16	6.70 (4.22; 12.15)	10.18 ± 18.78	6.10 (2.85; 9.80)	0.463
Glucose (mmol/L)	4.99 ± 0.84	4.90 (4.68; 5.30)	4.72 ± 0.97	4.70 (4.43; 5.28)	5.20 ± 0.66	5.00 (4.78; 5.48)	0.009
HOMA-IR	0.24 ± 0.51	0.14 (0.07; 0.25)	0.17 ± 0.12	0.13 (0.09; 0.24)	0.28 ± 0.65	0.14 (0.06; 0.25)	0.919
Adiponectin (ng/mL)	7841 ± 5381	5639 (4333; 9570)	10009 ± 6367	8282 (5144; 13660)	6433 ± 4136	4707 (4044; 8305)	0.002
Adiponectin/FAT in gram	0.43 ± 0.35	0.30 (0.18; 0.57)	0.41 ± 0.35	0.28 (0.17; 0.57)	0.44 ± 0.36	0.31 (0.18; 0.57)	0.861
Leptin (pg/mL)	10343 ± 12203	5579 (1866; 14725)	13900 ± 14637	8215 (2304; 22965)	8030 ± 9845	4570 (1829; 11983)	0.136
Leptin/FAT in gram	0.58 ± 1.01	0.39 (0.11; 0.72)	0.64 ± 0.44	0.69 (0.24; 0.97)	0.56 ± 1.19	0.33 (0.10; 0.57)	0.064
Leptin/adiponectin ratio	1.94 ± 2.86	0.69 (0.29; 2.46)	2.09 ± 2.95	1.03 (0.27; 2.58)	1.83 ± 2.84	0.64 (0.30; 2.35)	0.798
Resistin (ng/mL)	9.32 ± 3.35	8.85 (7.01; 11.20)	9.94 ± 2.99	10.00 (7.87; 11.83)	8.92 ± 3.55	8.67 (6.35; 10.63)	0.101

**Table 3:** Childhood cancer survivors according to BMI. Data are median and quartiles (1q; 3q) or mean ± SD.

	BMI > 25		BMI < 25		*p*
	*n* = 22		*n* = 53		
	Mean ± SD	Median (q1; q3)	Mean ± SD	Median (q1; q3)	
BMI (kg/m^2^)	28.65 ± 2.96	28.60 (26.08; 30.20)	21.62 ± 2.151	21.74 (20.11; 23.44)	<0.001
Age at study (years)	24.65 ± 2.91	25.21 (22.55; 26.30)	23.89 ± 3.72	23.34 (21.11; 25.72)	0.15
Follow-up after treatment (years)	12.71 ± 4.98	12.5 (7.6; 16.5)	11.46 ± 5.28	10.6 (6.85; 15.5)	0.41
Insulin (*μ*IU/mL)	16.03 ± 24.61	9.80 (7.60; 16.18)	6.23 ± 3.97	5.49 (2.84; 8.62)	<0.001
Glucose (mmol/L)	0.51 ± 0.14	0.51 (0.46; 0.59)	0.49 ± 0.05	0.49 (0.47; 0.52)	0.11
HOMA-IR	0.42 ± 0.86	0.23 (0.15; 0.34)	0.14 ± 0.10	0.12 (0.06; 0.19)	0.006
Fat	29336 ± 8874	28199 (23494; 35705)	15954 ± 9657	14182 (9545; 17527)	<0.001
FAT (%)	34.51 ± 9.12	33.50 (28.85; 39.20)	22.43 ± 8.07	20.65 (16.50; 27.00)	<0.001
Lean (g)	52792 ± 10682	55813 (41801; 62886)	49317 ± 9878	49742 (40681; 56797)	0.229
Lean/FAT	2.07 ± 1.22	1.87 (1.46; 2.33)	3.80 ± 1.72	3.63 (2.57; 4.78)	<0.001
Adiponectin (ng/mL)	6684 ± 3664	5733 (3944; 8346)	8345 ± 5941	5585 (4406; 10073)	0.312
Adiponectin/FAT in gram	0.26 ± 0.16	0.18 (0.13; 0.34)	0.52 ± 0.39	0.35 (0.26; 0.63)	0.003
Leptin (pg/mL)	14103 ± 16116	10246 (1716; 21653)	8707 ± 9819	5461 (2207; 13313)	0.525
Leptin/FAT in gram	0.42 ± 0.43	0.39 (0.07; 0.65)	0.67 ± 1.22	0.39 (0.21; 0.79)	0.217
Leptin/adiponectin ratio	2.63 ± 3.32	1.13 (0.26; 3.78)	1.63 ± 2.62	0.69 (0.33; 1.52)	0.439
Adiponectin/leptin ratio	1.99 ± 2.19	0.60 (0.26; 3.49)	2.70 ± 4.34	1.22 (0.62; 2.71)	0.357
Resistin (ng/mL)	9.27 ± 3.19	9.025 (6.51; 12.16)	9.34 ± 3.46	8.67 (7.14; 11.20)	0.906

**Table 4:** Patients characteristics by radiotherapy (RT). Data are median and quartiles (1q; 3q) or mean ± SD.

	RT		Non-RT		*p* value
	*n* = 50		*n* = 25		
	Mean ± SD	Median (q1; q3)	Mean ± SD	Median (q1; q3)	
Age at study (years)	24.65 ± 3.59	24.19 (22.51; 26.34)	23.04 ± 3.11	22.05 (20.91; 24.98)	0.06
Follow-up after treatment (years)	12.33 ± 5.17	12.30 (8.1; 16.2)	10.81 ± 5.2	9.8 (6.55; 14.6)	0.23
BMI (kg/m^2^)	23.79 ± 3.93	23.16 (20.80; 26.01)	23.79 ± 4.26	22.73 (20.89; 26.41)	0.74
WHR	0.85 ± 0.14	0.81 (0.77; 0.92)	0.84 ± 0.12	0.83 (0.74; 0.91)	0.39
FAT (%)	28.2 ± 11.3	28.5 (17.9; 37)	25.0 ± 8.10	21.7 (20.0; 31.8)	0.37
Lean (g)	48974 ± 9846	46740 (40849; 56463)	53566 ± 1050	55754 (46193; 60926)	0.11
Lean/FAT	3.07 ± 1.95	2.36 (1.59; 4.32)	3.26 ± 1.39	3.42(2.03; 3.79)	0.35
Insulin (*μ*IU/mL)	10.2 ± 18.0	6.10 (3.33; 11.6)	8.00 ± 5.10	6.97 (4.15; 9.48)	0.85
Glucose (mmol/L)	0.49 ± 0.09	0.49 (0.46; 0.54)	0.50 ± 0.04	0.49 (0.47; 0.52)	0.58
HOMA-IR	0.26 ± 0.61	0.13 (0.06; 0.25)	0.19 ± 0.14	0.16 (0.10; 0.24)	0.51
Adiponectin (ng/mL)	8395 ± 5440	6026 (4529; 10073)	6873 ± 5247	4855 (4223; 7466)	0.35
Adiponectin/FAT in gram	0.46 ± 0.36	0.34 (0.25; 0.60)	0.36 ± 0.33	0.26 (0.17; 0.35)	0.15
Leptin (pg/mL)	11499 ± 14382	5461 (1689; 18568)	8319 ± 6736	6463 (2747; 14093)	0.80
Leptin/FAT in gram	0.68 ± 1.24	0.40 (0.09; 0.79)	0.41 ± 0.31	0.33 (0.15; 0.65)	0.61
Leptin/adiponectin ratio	1.93 ± 3.00	0.60 (0.24; 1.92)	1.95 ± 2.66	1.02 (0.37; 2.87)	0.42
Resistin (ng/mL)	9.30 ± 3.67	8.77 (6.45; 11.41)	9.35 ± 2.80	8.85 (7.30; 10.63)	0.70

**Table 5:** Multivariate analyses of correlates of HOMA-IR in childhood cancer survivors. Coefficient of determination (*R* ^2^) was 0.54 for female and 0.31 for male subjects.

	Independent variable	Intercept (*α*)	Coefficient (*β*)	*t*	*p*	95% confidence interval	
Female	Leptin/adiponectin ratio	0.084	0.024	2.71	0.019	0.004	0.042
	Lean/FAT		−0.015	−0.56	0.585	−0.071	0.042
	BMI		0.003	0.30	0.770	−0.017	0.022

Male	Leptin/adiponectin ratio	−2.403	0.499	1.34	0.191	−0.026	0.126
	Lean/FAT		0.031	0.35	0.732	−0.151	0.212
	BMI		0.099	2.64	0.013	0.022	0.177
